# Association between gefitinib and hemorrhagic cystitis and severely contracted bladder: a case report

**DOI:** 10.1186/1471-2490-10-6

**Published:** 2010-02-26

**Authors:** Maki Arakawa, Kogenta Nakamura, Yoshiaki Yamada, Charles J Rosser, Motoi Tobiume, Hiroko Saito, Takaaki Hasegawa, Nobuaki Honda

**Affiliations:** 1 Department of Hospital Pharmacy, Aichi Medical University Hospital, Nagakute, Aichi 480-1195, Japan; 2 Department of Urology, Aichi Medical University School of Medicine, Nagakute, Aichi 480-1195, Japan; 3 Department of Urology, University of Florida, Gainesville, FL, USA

## Abstract

**Background:**

Gefitinib remains an excellent treatment option for patients with a variety of cancers, including non small cell lung cancer (NSCLC). However, clinicians must be aware of the potential of gefitinib to cause an inflammatory reaction in the skin, lungs and bladder.

**Case Presentation:**

We present a case on hemorrhagic cystitis and severaly contracted bladder in a patient with NSCLC on gefitinib.

**Conclusions:**

Further studies are needed to substantiate the association of gefitinib therapy with hemorrhagic cystitis and contracted bladder.

## Background

Epidermal growth factor receptor (EGFR) plays an important role in the growth, development and progression of non small cell lung cancer (NSCLC) [1]. Gefitinib (Iressa^®^) is an oral selective inhibitor of EGFR tyrosine kinase that has demonstrated efficacy in randomized double-blind phase III trials of the treatment of advanced NSCLC [2,3]. The side effect profile of gefitinib is quite acceptable, with diarrhea and skin rash, the most commonly reported side effects [2,3]. However, in addition to these main adverse events in Japan up to November 2008, a report by the pharmaceutical company to the Ministry of Health, Labour and Welfare indicated that 18 patients developed hematuria and 16 patients were diagnosed with hemorrhagic cystitis, which were induced by gefitinib, although the mechanism remains unclear. There is a report that severe hemorrhagic cystitis due to gefitinib was observed in only one of 25 patients [4]. We report a rare case in which microhematuria and lower urinary tract symptoms (LUTS) led to the diagnosis of hemorrhagic cystitis and a severely contracted bladder in a patient with NSCLC treated with gefitinib. The development of a severely contracted bladder can greatly impede urine storage, which can have severe effects on quality of life, necessitating major urinary reconstructive surgery.

## Case Presentation

**Figure 1:**
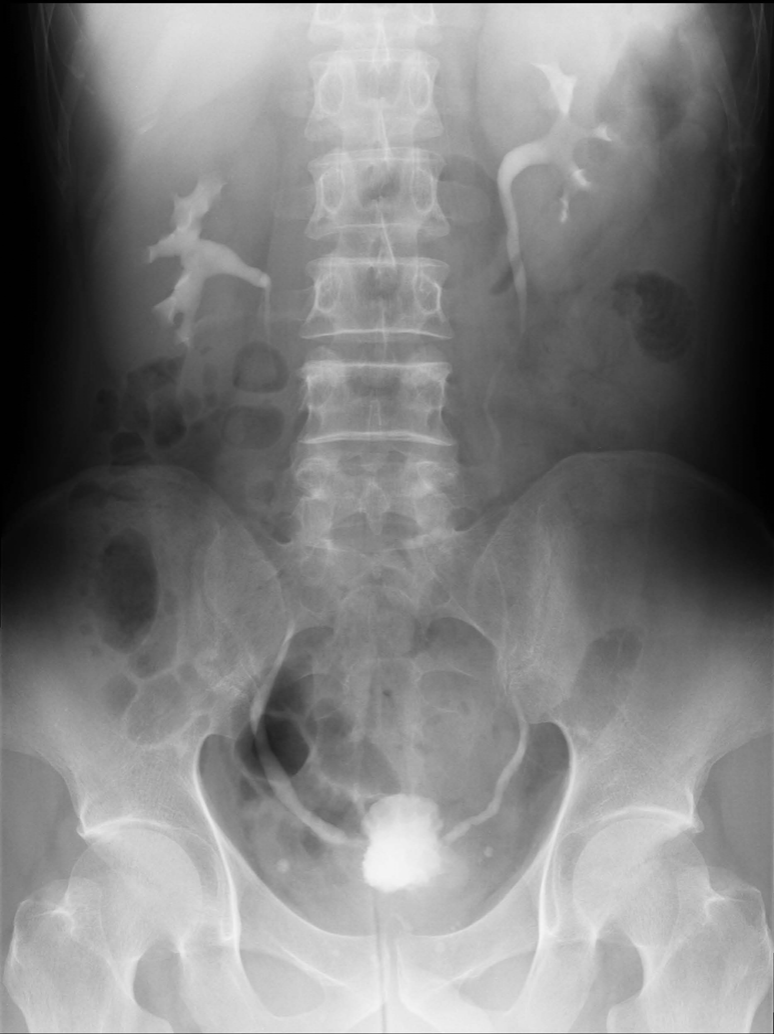
**Intravenous pyelography showed contracted bladder**.

**Figure 2:**
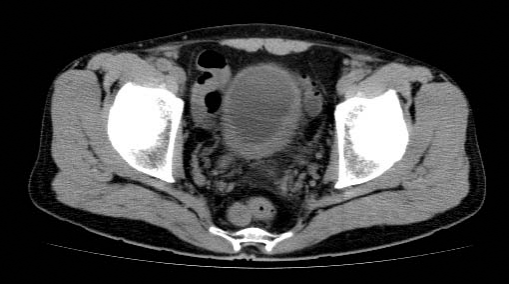
**Microscopic findings of bladder biopsy specimens revealed histological changes associated with hemorrhagic cystitis (× 10)**.

A 56-year-old Japanese man with biopsy-proven NSCLC was receiving active therapy with irinotecan (60 mg/m^2^) and cisplatin (60 mg/m^2^). Previously, the patient had had no significant genitourinary history. The combination of irinotecan and cisplatin was stopped because of persistent neuropenic fever and severe fatigue, and oral gefitinib (250 mg once a day) was initiated. Approximately two weeks later, severe LUTS, fever > 101.5°F and cough were noted. Urinalysis demonstrated fewer than 5 red blood cells/high power field, no white blood cells and no bacteria. Urine culture was negative. Sputum cultures showed chlamydial pneumonia, and the patient was started on moxifloxacin, with immediate improvement of cough but minimal improvement of fever, LUTS and hematuria. Over the next month, these symptoms persisted, together with deterioration of hepatic and renal function. Gefitinib therapy was halted. Empirically, ceftazidime was initiated and fever improved. Two weeks after stopping gefitinib, hepatic and renal function returned to normal. However, LUTS and microscopic hematuria continued despite negative urine cultures. The patient was referred to the Urology Department, and thorough investigation of hematuria (i.e., intravenous pyelography (IVP) assessing the kidneys and ureters and cystourethroscopy) was performed. IVP was normal except for a contracted, thickened bladder wall (Figure. 1). Cystourethroscopy revealed a small capacity bladder with erythematous lesions throughout the bladder. Because of the small capacity bladder, hydrodilation was performed. No discrete tumors or bladder calculi were noted. The prostatic urethra was unremarkable without trilobar hypertrophy or a prominent median lobe. Random bladder biopsies were obtained as well as urinary cytological examination. Urinary cytology was negative for malignancy. Histological examination of the bladder biopsies showed areas of denuded mucosa, submucosal edema, increased vascularity and white blood cell infiltration, indicative of hemorrhagic cystitis (Figure. 2). The symptoms improved over the ensuing four weeks.

## Discussion

Known causes of hemorrhagic cystitis include severe urinary tract infection, pelvic irradiation and alkylating anticancer agents. This report describes a case of hemorrhagic cystitis and a severely contracted bladder associated with gefitinib therapy. Our patient had no risk factors for the development of hemorrhagic cystitis and contracted bladder.

It is feasible that gefitinib therapy alone induced these inflammatory changes within the bladder, or it may have exacerbated non-bacterial cystitis presenting as LUTS and microscopic hematuria in this case of hemorrhagic cystitis and severely contracted bladder.

Hemorrhagic cystitis and the development of a severely contracted bladder have been related to lack of awareness of the risk of these side effects and belated discontinuation of gefitinib.

Gefitinib remains an excellent treatment option for patients with a variety of cancers. However, clinicians must be aware of the potential of gefitinib to cause hemorrhagic cystitis and contracted bladder, and thus must monitor for LUTS and hematuria in this cohort of patients. Further studies are needed to substantiate the association of gefitinib therapy with hemorrhagic cystitis and contracted bladder.

## Competing interests

The authors declare that they have no competing interests.

## Authors' contributions

MA drafted the report, and approved the final version of the manuscript. YY, MT and NH cared for the patient and approved the final version of the manuscript. HS and TH drafted the report and approved the final version of the manuscript. CJR reviewed the report and approved the final version of the manuscript. KN drafted the report, cared for the patient and approved the final version of the manuscript.

## Pre-publication history

The pre-publication history for this paper can be accessed here:

http://www.biomedcentral.com/1471-2490/10/6/prepub
